# 塞利尼索联合维奈克拉、阿扎胞苷方案诱导治疗复发难治急性髓系白血病的疗效及安全性观察

**DOI:** 10.3760/cma.j.cn121090-20231031-00241

**Published:** 2024-08

**Authors:** 丽娜 刘, 玉山 崔, 玉章 刘, 耀美 王, 谱 向, 利杰 梁, 以冉 李, 佰俊 房

**Affiliations:** 郑州大学附属肿瘤医院血液科，河南省肿瘤医院血液科，郑州 450008 Department of Hematology, the Affiliated Cancer Hospital of Zhengzhou University & Henan Cancer Hospital, Zhengzhou 450008, China

## Abstract

为探讨塞利尼索联合维奈克拉（VEN）、阿扎胞苷（AZA）在复发难治急性髓系白血病（R/R AML）患者中的疗效及安全性，纳入2022年5月至2023年5月在郑州大学附属肿瘤医院接受塞利尼索联合VEN、AZA方案的12例R/R AML患者，对其临床资料进行回顾性分析。12例患者中，完全缓解（CR）5例（41.7％），CR伴血液学不完全恢复1例（8.3％），部分缓解5例（41.7％）。达CR中位时间28（16～59）d。中位无病生存期为61（15～300）d。该方案的主要不良反应为血液学不良反应，无化疗相关死亡。塞利尼索联合VEN、AZA方案是治疗R/R AML的有效治疗手段。

## Abstract (en)

To determine the efficacy and safety of selinexor combined with venetoclax（VEN）and azactitidine（AZA）for patients with relapsed and/or refractory acute myeloid leukemia（R/R AML）. Twelve patients with R/R AML treated with selinexor plus VEN and AZA in the Affiliated Cancer Hospital of Zhengzhou University from May 2022 to May 2023 were included. Their clinical data were retrospectively analyzed. Among the 12 R/R AML patients, 5（41.7％）achieved complete remission（CR）, 1（8.3％）achieved CR with incomplete hematological recovery, and 5（41.7％）achieved partial remission. The median time to reach CR was 28（16–59）days. The median PFS was 61（15–300）days. The main adverse event of the regimen was hematological toxicity. No chemotherapy-related deaths were observed. The combination of selinexor plus VEN and AZA is an effective treatment for R/R AML patients.

近年来，国内外研究及临床实践均表明维奈克拉（VEN）联合阿扎胞苷（AZA）已成为老年、不适合高强度化疗初治及复发急性髓系白血病（AML）患者的新选择[1]，然而仍有许多复发患者无法达到再次缓解。输出蛋白1（XPO1）参与多种肿瘤抑制蛋白、致癌蛋白mRNA及生长调节因子的核输出，其高表达与肿瘤的发生、发展及不良预后相关[2]。塞利尼索是全球首款口服XPO1抑制剂，能够独立或与其他不同机制的药物发挥协同抗肿瘤活性，极具临床应用潜力。既往研究也证实了塞利尼索单药或联合方案在初治或复发AML患者中的疗效和安全性[3]–[4]。Luedtke等[5]研究发现塞利尼索与VEN联合使用可协同发挥抗AML活性，也有研究报道了塞利尼索与去甲基化药物能够协同发挥抗AML作用[6]–[7]。因此，我们尝试采用塞利尼索联合VEN+AZA方案诱导治疗复发难治AML（R/R AML），并对其临床疗效及不良反应进行初步观察。

## 病例与方法

1. 一般资料：回顾性分析2022年5月1日至2023年5月31日郑州大学附属肿瘤医院收治的12例应用塞利尼索联合VEN、AZA方案治疗的R/R AML患者，所有患者均根据2016年更新版WHO造血和淋巴组织肿瘤分类标准[8]诊断，经骨髓细胞形态学、免疫分型、细胞遗传学、分子生物学等检查确诊。遗传学危险度分层按照2021版中国AML诊疗指南[9]预后分层体系进行，分为预后良好、预后中等及预后不良组。所有患者均提供书面知情同意，本研究获河南省肿瘤医院医学伦理委员会批准（批件号：2018111）。

2. 诱导治疗：塞利尼索联合VEN+AZA方案：塞利尼索每次80 mg，第1、3、8、10天，口服；VEN 100 mg第1天，200 mg第2天，300 mg第3天，400 mg第4～28天，口服，每日1次；AZA 75 mg/m^2^，每日1次，第1～7天。可根据患者情况，对塞利尼索、VEN以及合并用药进行调整。对于伴有FLT3-ITD突变者，在三药基础上加用索拉非尼（每次400 mg，口服，每日2次）。若出现Ⅳ度骨髓抑制，同时合并重症感染时，可停用治疗药物。采用唑类抗真菌药物预防或治疗真菌感染时，调整VEN至200 mg，口服，每日1次。治疗第14天复查骨髓，若原始细胞<5％，继续口服VEN；若下降比例>50％但未达<5％，继续口服VEN；若无效，更改方案再诱导治疗。达完全缓解（CR）后行巩固治疗，有合适供者且符合要求者行异基因造血干细胞移植（allo-HSCT）。

3. 治疗期间不良事件的发生及处理原则：所有不良事件按照美国国家癌症研究所（NCI）通用不良事件术语标准5.0版进行报告和分级。治疗期间定期监测患者血常规、肝肾功能、电解质、心肌酶谱及凝血功能，当患者出现咳嗽、咳痰、寒战、发热等感染症状时，完善痰培养、血培养及降钙素原（PCT）检查，必要时完善胸部CT等影像学检查，经验给予广谱抗感染药物，同时积极给予对症支持治疗；当患者出现骨髓抑制时，给予细胞刺激因子、血制品输注等对症支持治疗。

4. 疗效评估：按照2022年欧洲白血病网（ELN）AML诊疗指南进行疗效评估，CR：骨髓原始细胞<5％，原始细胞内不含有棒状小体（Auer rod）；外周血无原始细胞；无髓外白血病；外周血中性粒细胞绝对计数>1.0×10^9^/L，且PLT>100×10^9^/L。CR伴血液学不完全恢复（CRi）：外周血中性粒细胞绝对计数<1.0×10^9^/L或PLT<100×10^9^/L，其他满足CR标准。部分缓解（PR）：化疗后骨髓原始细胞比例减少至少50％，且下降到5％~25％，外周血细胞计数正常。总体反应率（ORR）定义为患者在治疗后达到CR、CRi、PR的比例。未缓解（NR）：诱导治疗后未获得CR或PR。无病生存（DFS）期指患者达CR至复发、死亡或随访截止的时间。

5. 随访：随访截至2023年5月31日。中位随访时间127（43～355）d。末次随访疾病状态，包括持续缓解、复发、失访。随访方式包括电话随访、门诊复查、查阅病历。

6. 统计学处理：采用SPSS19.0软件进行统计学分析。治疗前后骨髓幼稚细胞比例比较采用配对*t*检验，*P*<0.05为差异有统计学意义。

## 结果

1. 临床特点：12例R/R AML患者中，男6例，女6例，中位年龄56（20～74）岁；6例为原发性AML，6例为继发性AML（其中5例由骨髓增生异常综合征转化，1例由原发性骨髓纤维化转化）；4例为难治AML（2个疗程诱导治疗未达CR），8例为复发AML（其中4例同时伴中枢神经系统浸润）；10例患者治疗前存在感染、心功能不全等并发症；治疗前中位骨髓幼稚细胞比例为49.6％（22.0％～87.5％）；12例患者中预后中等者8例，预后不良者4例；5例患者存在AZA的单暴露，4例患者存在VEN和AZA的双暴露。

2. 疗效评价：截至2023年5月31日，所有患者均顺利完成诱导治疗。12例R/R AML患者接受塞利尼索联合VEN+AZA方案治疗后ORR为91.7％。治疗后骨髓幼稚细胞比例明显下降［治疗后中位值5.4％（0.5％～26.0％），基线时中位值49.6％（22.0％～87.5％），*P*<0.01］。5例CR，1例达CRi，5例达PR，达CR中位时间28（16～59）d。上述患者中位DFS时间为61（15～300）d。具体疗效及转归见表1。

**表1:** 塞利尼索联合VEN+AZA方案治疗的12例难治复发AML患者的疗效及转归

例号	性别	年龄（岁）	2016 WHO分型	疾病状态	前期治疗	合并症	疗效	转归
1	男	20	AML非特指型	原发难治	IA、HAG	肺炎	CR	巩固治疗中，持续CR 2个月余，已行HLA配型
2	男	54	AML伴既往MDS病史；伴中枢浸润	2线复发	AZA+AG、AZA+亚砷酸、AZA+HAG	肺炎	PR	诱导治疗中，持续PR 2个月余
3	女	32	AML伴重现性基因异常；伴中枢浸润	2线复发	ATRA+DNR、DA、ATRA+亚砷酸、HAG+ATRA、ATRA+亚砷酸	无	CR	巩固治疗中，持续CR 3个月余
4	男	51	AML非特指型	原发难治	CAG、HAG	肺炎、脓毒血症、低蛋白血症	CR	诱导治疗达CR后15 d，出现肺部感染加重，放弃治疗
5	女	64	AML非特指型	原发难治	CAG、AZA+HAG	肺炎、泌尿系感染	CR	巩固治疗中，持续CR 10个月余
6	女	56	AML非特指型	2线复发	IA、DA、HD-Ara-C、AZA+CAG、AZA+AG	肺炎	PR	诱导治疗中，仍未CR
7	男	68	AML伴既往MPN病史；伴中枢浸润	1线复发	VEN+AZA	肺炎	PR	诱导治疗达PR 1个月后失访
8	男	72	AML伴既往MDS病史	4线复发	AZA+CAG、AZA+AG、AZA+亚砷酸、DA、DAC+HAG、AZA+AG、干白沙	肺炎、肠道感染	CR	巩固治疗中，持续CR 4个月余
9	女	35	AML伴既往MDS病史；伴中枢浸润	3线复发	AZA+CAG、AZA+IA、AZA+AG、AZA+VEN+HAG	肺炎、肛周感染	PR	同方案再次诱导治疗1个周期后，行同胞全相合allo-HSCT
10	女	56	AML伴既往MDS病史	原发难治	AZA+CAG、AZA+VEN+HAG	肺炎	NR	诱导治疗中，已行HLA配型
11	女	75	AML伴重现性基因异常	3线复发	CAG、AZA+CAG、AZA+IA、IA、干白沙	心功能不全，间歇性房颤	PR	治疗2个周期后，失访
12	男	59	AML伴既往MDS病史	1线复发	AZA+VEN	无	CRi	巩固治疗中，持续CR 5个月余

**注** AML：急性髓系白血病；MDS：骨髓增生异常综合征；MPN：骨髓增殖性肿瘤；VEN：维奈克拉；AZA：阿扎胞苷；Ara-C：阿糖胞苷；DNR：柔红霉素；ATRA：全反式维甲酸；HAG：高三尖杉酯碱+Ara-C+G-CSF；IA：盐酸伊达比星+Ara-C；AG：Ara-C+G-CSF；CAG：盐酸阿柔比星+Ara-C+G-CSF；DA：柔红霉素+Ara-C；干白沙：干扰素+沙利度胺+白细胞介素2；HD-Ara-C：大剂量Ara-C；CR：完全缓解；CRi：完全缓解伴血细胞不完全恢复；PR：部分缓解；NR：未缓解；allo-HSCT：异基因造血干细胞移植

3. 不良反应：治疗过程中12例患者均出现3～4级血液学不良反应，包括白细胞减少（100％）、中性粒细胞减少（100％）、贫血（91.7％）、血小板减少（100％）。中性粒细胞缺乏的中位时间16.5（3～38）d，中位HGB最低值59.5（46～82）g/L，中位PLT最低值9（1～17）×10^9^/L，予以G-CSF及输血对症支持治疗后好转。主要的非血液学不良反应为1～2级的乏力（91.7％）、无症状低钠血症（75.0％），所有患者给予甲地孕酮及托烷司琼预防胃肠道反应，41.7％患者发生恶心，16.7％患者发生呕吐。可见3级以上感染相关的不良反应，包括肺炎（33.3％）、肛周感染（8.3％），予以抗细菌联合抗真菌治疗好转。所有患者未出现肿瘤溶解综合征。

## 讨论

对于高龄、合并严重并发症等不适合强化疗的R/R AML患者，目前治疗手段有限，其预后极差。VEN+AZA治疗初治AML取得了喜人疗效[10]，同时VEN联合去甲基化药物被推荐用于R/R AML患者[11]。然而，VEN+AZA耐药的出现仍是临床治疗中亟需解决的难题。据报道，经VEN+AZA治疗后获得CR/CRi患者，约52％出现疾病进展或形态学复发[12]。且VEN联合去甲基化药物治疗失败的AML患者，中位生存时间仅约2.9个月[13]。因此，VEN+AZA联合其他靶向药（如吉瑞替尼、艾伏尼布）或其他新药等组成三联方案或许是一种新的治疗选择。

塞利尼索是首款XPO1抑制剂。XPO1是核转运蛋白家族中的一员，主要负责多种肿瘤抑制因子、生长调节因子等物质的核输出。塞利尼索通过可逆性结合XPO1，导致肿瘤抑制蛋白在细胞核内积累，引发肿瘤细胞凋亡[14]。研究发现，XPO1高表达与AML较短的生存期相关[15]。据报道，塞利尼索单药及联合化疗可有效降低AML患者外周血及骨髓原始幼稚细胞比例，但塞利尼索单药治疗ORR及CR率均较低[3]。国内外不同中心分别进行了塞利尼索联合大剂量阿糖胞苷和米托蒽醌[16]、DA（柔红霉素+阿糖胞苷）[17]、FLAG（氟达拉滨+阿糖胞苷）[18]等方案的尝试。虽然AML患者的CR率得到明显提高，但因较强的化疗毒性，高龄、合并严重并发症等患者的生存并未获益。因此，寻求高效低毒的治疗方案是改善R/R AML患者生存的重点。

研究发现，塞利尼索联合VEN通过调控DNA损伤修复协同发挥抗白血病效应[19]。一项评估塞利尼索联合VEN在R/R AML和非霍奇金淋巴瘤中的有效性及安全性的临床试验正在进行中（NCT02485535）。体外研究证实，塞利尼索联合AZA可致MDS细胞周期阻滞，并诱导抑癌蛋白p53在细胞核中积聚进而促使MDS细胞的凋亡[20]。一项Ⅱ期临床研究揭示对去甲基化药物耐药的MDS或AML患者，仍可从塞利尼索中获益[21]。这提示塞利尼索或许可逆转MDS或AML患者对去甲基化药物的耐药。此外，塞利尼索联合AZA通过靶向XPO1/eIF4E/c-MYC通路协同发挥抗白血病效应[7]。据国内一项单中心回顾性研究报道，塞利尼索联合去甲基化药物在VEN暴露的R/R AML中取得良好的疗效[22]。

鉴于塞利尼索与VEN、AZA的协同抗白血病效应，我们尝试采用塞利尼索联合VEN+AZA方案诱导治疗12例R/R AML患者。诱导治疗后，5例达到CR（4例原发AML，1例MDS转化），1例达CRi（MDS转化），5例达PR（2例原发AML，2例MDS转化，1例MPN转化），1例（MDS转化）诱导NR。相较于MDS转化患者，原发AML患者缓解率更高。4例中枢神经系统浸润患者，其中1例为原发AML伴中枢神经系统浸润，诱导治疗后达CR；另外3例为转化AML伴中枢神经系统浸润，诱导治疗后达PR，但脑脊液形态及微小残留病均转阴。这提示我们，对于伴中枢神经系统浸润、化疗耐受性差的AML患者，应用能够透过血脑屏障的小分子靶向药物是一种较好选择。

纳入研究的12例患者中，5例患者存在AZA的单暴露：例5和例8取得了CR，例2、6、11取得了PR；4例患者存在VA的双暴露，例12取得了CRi，例7和例9取得了PR。这提示既往VEN和（或）AZA暴露的R/R AML患者仍可从塞利尼索中获益，值得进一步关注。

10例治疗前存在重症感染、心功能不全患者均顺利完成诱导治疗，且治疗期间新发感染率较常规化疗低，提示该方案安全性良好。治疗期间应密切监测VEN血药浓度、骨髓抑制等情况，及时调整用药剂量。塞利尼索联合VEN+AZA方案的给药剂量、给药顺序等需进一步优化以提效减毒。

综上所述，塞利尼索联合VEN+AZA方案对于高龄、合并严重并发症等不适合强化疗的R/R AML患者安全、有效，并为桥接allo-HSCT争取时机，值得进一步探索。
